# The rehydration transcriptome of the desiccation-tolerant bryophyte *Tortula ruralis*: transcript classification and analysis

**DOI:** 10.1186/1471-2164-5-89

**Published:** 2004-11-16

**Authors:** Melvin J Oliver, Scot E Dowd, Joaquin Zaragoza, Steven A Mauget, Paxton R Payton

**Affiliations:** 1 Cropping Systems Research Laboratory, USDA-ARS, 3810 4^th ^St, Lubbock, TX, USA

## Abstract

**Background:**

The cellular response of plants to water-deficits has both economic and evolutionary importance directly affecting plant productivity in agriculture and plant survival in the natural environment. Genes induced by water-deficit stress have been successfully enumerated in plants that are relatively sensitive to cellular dehydration, however we have little knowledge as to the adaptive role of these genes in establishing tolerance to water loss at the cellular level. Our approach to address this problem has been to investigate the genetic responses of plants that are capable of tolerating extremes of dehydration, in particular the desiccation-tolerant bryophyte, *Tortula ruralis*. To establish a sound basis for characterizing the *Tortula *genome in regards to desiccation tolerance, we analyzed 10,368 expressed sequence tags (ESTs) from rehydrated rapid-dried *Tortula *gametophytes, a stage previously determined to exhibit the maximum stress induced change in gene expression.

**Results:**

The 10, 368 ESTs formed 5,563 EST clusters (contig groups representing individual genes) of which 3,321 (59.7%) exhibited similarity to genes present in the public databases and 2,242 were categorized as unknowns based on protein homology scores. The 3,321 clusters were classified by function using the Gene Ontology (GO) hierarchy and the KEGG database. The results indicate that the transcriptome contains a diverse population of transcripts that reflects, as expected, a period of metabolic upheaval in the gametophyte cells. Much of the emphasis within the transcriptome is centered on the protein synthetic machinery, ion and metabolite transport, and membrane biosynthesis and repair. Rehydrating gametophytes also have an abundance of transcripts that code for enzymes involved in oxidative stress metabolism and phosphorylating activities. The functional classifications reflect a remarkable consistency with what we have previously established with regards to the metabolic activities that are important in the recovery of the gametophytes from desiccation. A comparison of the GO distribution of *Tortula *clusters with an identical analysis of 9,981 clusters from the desiccation sensitive bryophyte species *Physcomitrella patens*, revealed, and accentuated, the differences between stressed and unstressed transcriptomes. Cross species sequence comparisons indicated that on the whole the *Tortula *clusters were more closely related to those from *Physcomitrella *than Arabidopsis (complete genome BLASTx comparison) although because of the differences in the databases there were more high scoring matches to the Arabidopsis sequences. The most abundant transcripts contained within the *Tortula *ESTs encode Late Embryogenesis Abundant (LEA) proteins that are normally associated with drying plant tissues. This suggests that LEAs may also play a role in recovery from desiccation when water is reintroduced into a dried tissue.

**Conclusion:**

The establishment of a rehydration EST collection for *Tortula ruralis*, an important plant model for plant stress responses and vegetative desiccation tolerance, is an important step in understanding the genome level response to cellular dehydration. The type of transcript analysis performed here has laid the foundation for more detailed functional and genome level analyses of the genes involved in desiccation tolerance in plants.

## Background

The cellular response of plants to water deficits has both economic and evolutionary importance directly affecting plant productivity in agriculture and plant survival in the natural environment. Ramanathan [1] has argued, based on predictions of global environmental changes, that developing crops which are more tolerant to water deficits while maintaining productivity, will become a critical requirement in the early part of the 21^st ^century. Understanding how plant cells tolerate water loss is a vital prerequisite for developing strategies for improving tolerance of, and biomass/seed production under drought conditions.

In the last decade the genes induced by water-deficit stress have been successfully enumerated in plants that are relatively sensitive to cellular dehydration, in particular *Arabidopsis thaliana *[2-6]. In addition, the mechanisms by which the genetic response to water deficit is controlled by abscisic acid (ABA)-dependent and independent pathways have also been extensively elucidated [6,7]. However, even with the recent addition of in-depth examination of gene expression patterns using *Arabidopsis *microarrays [8,9] we have little functional knowledge of the genes that respond to water deficits. Of critical importance is the question of which of the genes identified as responding to water deficits actually have an adaptive role in establishing tolerance, in particular tolerance of cellular dehydration, and which are genes that are only responding to the injury incurred by the imposition of the stress. Injury may induce, or repress, specific genes that are not involved in promoting adaptation to cellular dehydration. An indication that at least some of the genes have an adaptive function in dehydration tolerance derives from the observation that they are expressed in tissues that acquire desiccation tolerance, the extreme manifestation of dehydration tolerance, such as in maturing seeds and in leaves of desiccation-tolerant plants during drying [3,10,11]. However, in order to fully address the question of the adaptive importance of genes involved in responses to cellular dehydration it is necessary to gain an evolutionary perspective of the involvement of a gene in dehydration tolerance mechanisms. To this end we have established an ongoing comparative genomics program to study the genetic responses to dehydration in species that span the evolution of dehydration (desiccation) tolerance mechanisms within the land plants. Here we present an analysis of ESTs derived from the desiccation-tolerant bryophyte *Tortula ruralis *[Hedw.] Gaertn. Meyer & Scherb., that is representative of the primitive genetic strategy for the acquisition of desiccation-tolerance [see [12,13]].

Desiccation tolerance, the ability to recover from the almost complete loss (90%) of protoplasmic water, is a phenomenon common in the reproductive structures of green plants: pollen, spores and seeds. However, the ability to survive vegetative desiccation is a demonstrable but uncommon occurrence in the plant kingdom [13-18]. Within the flowering plants there are only approximately 300 species of flowering plants that are known to tolerate vegetative desiccation [16,17].

Recent physiological phylogenetic analyses indicate that vegetative desiccation tolerance was primitively present in the bryophytes (the basal-most living clades of land plants), but was lost in the evolution of tracheophytes. Desiccation-tolerant bryophytes are found worldwide and occupy a variety of ecological niches, most of which could, during some period of the year, be considered as extreme either on a macro or microhabitat level. In most cases the extremes that these plants experience are both in water availability and temperature [19-23].

Desiccation-tolerant bryophytes, because of their simple architecture, have few, if any, morphological (or indeed physiological) characteristics or adaptations that can limit water loss or regulate plant temperature. As a result of this, the internal water content of their photosynthetic tissues rapidly equilibrates to the water potential of the environment once free water is lost from the surface of the plant. This in turn means that these plants experience drying rates that are much faster than those experienced by their more complex pteridophyte or angiosperm counterparts. In fact, the drying rates that desiccation-tolerant bryophytes experience are generally lethal to desiccation-tolerant ferns and flowering plants [14]. The rapid equilibration of protoplasmic water potential with that of the environment in bryophyte tissues appears to demand a type of desiccation tolerance that is significantly different from that exhibited by desiccation tolerant angiosperms [15]. Rather than acquiring desiccation tolerance in response to a dehydration event as seen in *Craterostigma plantagineum*, *Sporobolus stapfianus*, and other desiccation-tolerant angiosperms, desiccation-tolerant bryophytes appear to express this trait constitutively [15,24]. This form of desiccation tolerance is considered the most primitive of those that have received attention so far [13]. In this type of tolerance the primary response to a desiccation event, at least at the level of gene expression, occurs after the fact, during the first hour or two following rehydration. This has led to the suggestion that a major component of the mechanism of desiccation tolerance in bryophytes is a rehydration-induced cellular repair response [15,24]. The implication is that although cellular protection and hence desiccation tolerance is constitutive, it is not sufficient to prevent some damage from occurring (or being manifested) upon rehydration, and thus repair processes are needed and induced when water returns to the protoplasm of the cells.

The repair aspect of the mechanism of desiccation tolerance in these plants, although demonstrated to be a major component of tolerance, is difficult to detail and characterize. Most work has focused on the proteins whose synthesis is induced immediately upon rehydration of desiccated gametophytic tissue. Early work [25] established the ability of *T. ruralis *and other mosses to rapidly recover synthetic metabolism when rehydrated. The speed of this recovery was inversely dependent upon the rate of prior desiccation: the faster the rate of desiccation, the slower the recovery. In addition, although the pattern of protein synthesis in the first two hours of rehydration of *T. ruralis *is distinctly different from that of hydrated controls, novel transcripts were not made in response to desiccation [26]. Hence it was suggested that *T. ruralis *responds to desiccation by an alteration in protein synthesis upon rehydration that is in large measure the result of a change in translational control. Changes in transcriptional activity were observed for nearly all transcripts studied [27] but did not result in a qualitative change in the transcript population during desiccation or rehydration. It thus appears that *T. ruralis *relies more upon the activation of pre-existing repair mechanisms for desiccation tolerance than it does on either pre-established or activated protection systems.

In a detailed study of the changes in protein synthesis initiated by rehydration in *T. ruralis*, Oliver [26] demonstrated that during the first two hours of hydration the synthesis of 25 proteins is terminated, or substantially decreased, and the synthesis of 74 proteins is initiated, or substantially increased. Controls over changes in synthesis of these two groups of proteins, the former termed hydrins and the latter rehydrins, are not mechanistically linked. It takes a certain amount of prior water loss to fully activate the synthesis of rehydrins upon rehydration. RNA blots revealed that several rehydrin transcripts accumulate during slow drying [28,29] at a time when it is assumed that transcriptional activity is rapidly declining. These transcripts do not accumulate during rapid desiccation, nor is their accumulation during slow drying associated with an increase in endogenous ABA accumulation. ABA is undetectable in this moss [[30], M. J. Oliver, unpubl data], and *T. ruralis *does not synthesize specific proteins in response to applied ABA. The accumulation of these transcripts was postulated to be the result of an increase in mRNA stability brought about by the removal of water from the cells [27]. Recent studies clearly demonstrate that these transcripts are sequestered in the dried gametophytes in mRNP particles [29] and that this results in the change in their stability. The implication from this work is that the sequestration of mRNAs required for recovery hastens the repair of damage induced by desiccation or rehydration and thus minimizes the time needed to restart growth upon rehydration.

The major question arising from these studies concerns the identity of rehydrins and what possible functions and roles they may play in regards to the response to desiccation and rehydration and to desiccation tolerance *per se*. We have some limited knowledge of the functions (or postulated functions) of a few of the rehydrins from classical molecular analyses, [31,32] and from a small-scale EST collection analysis [33]. However, there is an obvious need to extend this base and to develop testable hypotheses that will help us to elucidate the metabolic and genetic mechanisms that control the recovery and repair of dried plant cells and their role in the development and evolution of desiccation tolerance. To gain an appreciation of the number of possible rehydrin genes and the range of possible functions encompassed by their expression we have initiated a genomics level analysis of gene expression during the recovery of *Tortula *gametophytes from the desiccated state. The first step in this process was to establish an EST collection that is representative of the transcripts available to the moss during the first few hours following rehydration. In this report we present a bioinformatic analysis of 10, 368 ESTs from the early phases of recovery following rehydration of rapidly-dried *Tortula ruralis *gametophytes; rapid dried gametophytes were chosen in order to maximize the recovery and repair response upon rehydration. The bioinformatics approach we have taken is based on that used by McCarter et al., [34] to conduct a comprehensive analysis of 5,700 *Meloidogyne incognita *L2 ESTs, and includes cluster analyses, transcript abundancy estimations, and functional classifications based on InterPror domains, Gene Ontology hierarchy, and KEGG biochemical classifications. The overall goal was to gain an appreciation of the *Tortula *transcriptome during the time period following rehydration when the desiccation driven alteration in gene expression is at its peak. During this period we hypothesize the processes of cellular repair and recovery are the main focus of the metabolism of the gametophytic cells.

## Results and discussion

Ten thousand three hundred and sixty eight individual cDNA clones were selected from a *Tortula ruralis *rehydration library and subjected to single-pass 5' directional sequencing to generate 10,368 primary ESTs of which 9,159 (88%) passed through quality control, vector trimming, E. coli contamination, and cloning artifact removal. The 9,159 ESTs averaged 648 nucleotides in length and totaled 5.93 million nucleotides submitted to Genbank. These submitted ESTs form the basis of the subsequent transcriptome analysis utilizing the High Throughput-Gene Ontology-Genome Annotation Toolkit (HT-GO-GAT), a software developed by S.E. Dowd (unpublished).

### Cluster analysis

**Figure 1:**
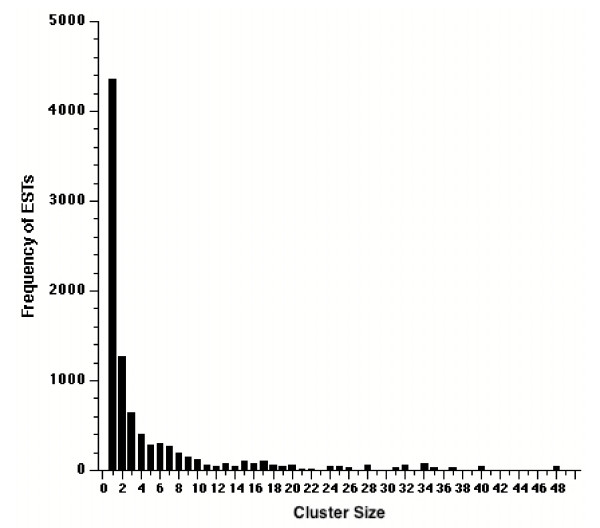
Histogram of distribution of ESTs by cluster size.

Utilizing the assembly algorithms incorporated in the SeqManII software (part of the DNASTAR suite from DNASTAR Inc, Madison WI), the 9,159 ESTs were grouped into contigs and clusters by establishing assembly stringencies that generated groupings defined by the analysis protocols of McCarter et al., 2003 [34]. Contigs contain EST members that appear to originate from single transcripts whereas clusters are assemblies of ESTs that could represent transcripts from the same gene but alternate splice isoforms, or in the case of *Tortula *ESTs, which derive from a population of individual gametophytes, alleles of the same gene. The 9,159 ESTs formed 7,272 contigs and 5,563 clusters, both of which exhibit an average size of 669 nucleotides. However, the longest sequence increased from 1,575 nucleotides for contigs to 2,229 nucleotides for clusters. Clusters varied in content from a single EST (singletons) in 4,362 cases (78%) to 48 ESTs for a single cluster (Figure 1). The elimination of redundancy during contig building and cluster formation reduced the total number of nucleotides for further analyses from 5.93 million to 4.87 million (contigs) and 3.71 million (clusters). Overall the 9,159 ESTs potentially represent 5,563 genes, a discovery rate of 60%, with 47.6% of the ESTs as singletons. This is an overestimation of gene discovery since several non-overlapping clusters can represent a single gene and from our blast search data this appears to be a possibility, at least in the case of clusters 121 and 204 that appear to independently represent a gene that has a weak similarity to the LEA protein of *Caenorhabditis elegans*. Even though the ESTs derive from a non-normalized library 96.5% of the clusters still have 5 or fewer EST members.

### Transcript abundance

The consensus Cluster sequences were subjected to a BLASTx style search of a custom curated non-redundant database derived from UNIPROT and annotated according to the degree of similarity of the cluster to the highest scoring match in the database of known gene sequences. The degree of similarity was based on the quality of the BLASTx statistical outputs as well as a visual inspection of the aligned sequences between the query and the target. Clusters that generated an HSP-bit score below 70 and or E values higher than 10^-7 ^were assessed manually for all possible alignments taking into account the alignment length, number of identical matches, gaps, and positive replacements. Utilizing these criteria we were able to annotate 3,321 (59.7 %) of the 5,563 clusters by their similarity to known genes within the database. This also meant that 2,242 of the clusters, or 40.3%, represent sequences that have no known counterpart in the public databases and that we categorize as unknowns.

Table 1 lists the 30 most abundant EST clusters derived from the *Tortula *rehydration EST collection. These transcripts only account for 8.4% of the generated ESTs, however. Ten of the most abundantly represented transcripts encode proteins that do not match any of the sequences in the databases searched in this study and are designated as unknowns. Seven abundant transcripts appear to encode proteins that belong to a class of proteins known as Late Embryogenesis Abundant (LEA) proteins, although the relatively low HSP bit scores and high E-values for most of these BLASTx matches has to be considered as a caveat in this assessment. LEA proteins have long been ascribed a protective role for cells that are experiencing dehydration [11,35]. This is a conclusion drawn on the strong correlation between LEA transcript accumulation and water loss, rapid decline in transcript levels upon rehydration, and especially as LEA gene expression relates to the programmed desiccation stage of seed maturation. If one can conclude that LEA protein transcripts are abundant in the rehydration transcriptome of *Tortula ruralis*, which is consistent with some of our earlier findings [32], then it is also possible that these proteins are involved in either protection of cellular integrity during the initial phases following rehydration when cell disruption is apparent in bryophytes [36], or are actively involved in the restoration of cells damaged by a desiccation event. This difference in the response of LEA gene expression between *Tortula *and what has been reported for angiosperms may also be a reflection of what we believe to be a more primitive mechanism of dehydration tolerance, and perhaps a more primitive role for and control of LEA gene expression. Other abundant transcripts appear to fall into the membrane transport (aquaporin, cysteine rich proteins, channel and pore proteins) and proteins that can be associated with plant stress events (metallothionine, esterase, and rubredoxin). The Early Light Inducible Protein A (ELIP-A) transcript is the only member of the abundant transcripts that we have previously reported [33] as belonging to the group of proteins we have termed, rehydrins [31]. These proteins have been suggested to be synthesized in response to stress-induced photo-damage within the *Tortula *chloroplast and may play a protective or repair function for the photosynthetic apparatus [37].

**Table 1:** Thirty most abundant transcripts in the *Tortula *rehydration library.

Length	#ESTs in Cluster	Description	TrEMBL Accession #	Bit Score	E-value
962.0	48	Unknown			
1102.0	40	ABA-inducible protein WRAB1 (Cold-responsive LEA/RAB-related COR protein)	Q9XFD0	63.93	2.88E-09
1000.0	37	Caenorhabditis elegans CE-LEA	O16527	75.87	6.51E-13
889.0	35	Unknown			
1000.0	34	TspO tryptophan rich sensory protein homologue	Q92ZA7	127.87	1.43E-28
994.0	34	Unknown			
975.0	32	Hydrophobic LEA-like protein (Oryza sativa)	Q9ZRF8	100.91	1.84E-20
964.0	32	Unknown			
1201.0	31	Cysteine rich protein (ion transport)	Q24977	50.45	3.86E-05
1051.0	28	Late embryogenesis abundant (LEA) protein 76 (Brassica napus)	P13934	51.60	1.42E-05
683.0	28	Cysteine-rich non-metallothionein-protein	Q24774	47.75	1.05E-04
1078.0	26	Early light-inducible protein ELIPA (Tortula ruralis)	Q8RYB6	404.06	1.24E-111
956.0	25	Rb7 (Fragment)-MIP/Aquaporin	O04179	215.70	3.62E-55
732.0	25	Stress-inducible membrane pore protein	Q93Z87	88.58	6.21E-17
1043.0	24	Ribosomal-protein-alanine acetyltransferase	Q8IAE2	116.32	4.73E-25
942.0	24	Unknown			
902.0	22	Caenorhabditis elegans CE-LEA	O16527	50.83	2.02E-05
1043.0	21	Protein DR1172-LEA type 1 family	Q9RV58	55.45	1.01E-06
984.0	20	Unknown			
661.0	20	Core protein (Pisum sativum) amino acid-selective channel protein	Q41050	79.34	3.13E-14
514.0	20	Pyrus pyrifolia Metallothionein-like protein	Q9LUX2	57.38	7.04E-08
1316.0	19	Hypothetical protein K08H10.2a	Q9XTH4	92.82	8.12E-18
996.0	19	Unknown			
982.0	18	Putative late embryogenesis abundant protein (Arabidopsis)	Q9LF88	53.91	2.63E-06
910.0	18	Unknown			
790.0	18	Gb|AAF26109.1 (Hypothetical protein) Arabidopsis	Q9FFJ0	125.56	5.31E-28
1717.0	17	Lanatoside 15'-O-acetylesterase precursor (Foxglove)	O82681	165.62	1.39E-39
1000.0	17	Unknown			
1000.0	17	P0577B11.21 protein (Oryza sativa) Rubridoxin-like	Q84SC5	60.08	3.85E-08
957.0	17	Unknown			

Although transcript abundance may reflect the metabolic or physiological needs of the moss during the rehydration phase of a wet/dry/wet cycle it would be more desirable to know how these transcripts are recruited and utilized by the translational machinery to make the proteins that actually contribute to the recovery of the gametophytes following rehydration. Such information is beyond the scope of this analysis but the identification and isolation of the Clusters that represent the *Tortula *rehydration transcriptome does represent the first major step for such a pursuit.

### Functional classification of transcripts

Physiological, biochemical, and molecular data all point towards an active period of cellular activity, presumably for repair and recovery from desiccation induced damage, during the first two hours following rehydration of dried gametophytes [13,15]. The identity of the more abundant transcripts (Table 1.) does provide some insight into the nature of the metabolic activity that is associated with rehydration, at least it gives an indication of what metabolic processes may be of prime concern as the plant recovers from desiccation. However, the functional classification of the *Tortula *transcripts present during the initial phases following rehydration using the Gene Ontology (GO) classification system paints a broader view of the possible metabolic activity of the gametophytic cells at this time. This does, of course, come with the understanding that these are transcript based analyses and do not directly reflect protein levels which would offer a more definitive assessment of the metabolic capability of the cells during rehydration.

Functional classification of the *Tortula *rehydration transcripts was achieved by matching the *Tortula *clusters to characterized protein domains in a combined protein database, using HT-GO-GAT (Materials and Methods), which allowed us to assign GO terms to each cluster. The assignment of GO terms made it possible to place the clusters into the GO hierarchy which can be viewed by use of an AmiGo browser. Of the 3, 321 clusters that exhibited significant similarity to known genes in the public databases, 2,203 (66% of annotated or 40% of all clusters) represent genes that contain conserved protein domains that have known biochemical and physiological functions in other organisms and map to the GO hierarchy. The GO representations for the *Tortula *rehydration clusters are presented in Tables 2 through 4. The representations are segregated into the three main organizing principles of GO: biological process (Table 2), cellular component (Table 3), and molecular function (Table 4). A complete listing of the GO mappings is available on our website at .

**Table 2:** Gene ontology (GO) mappings : Biological processes 1673 clusters

Categories and subcategories	Representation			% of Total		
Cellular Processes	409			24.5		
Cell Communication		75			4.5	
Signal Transduction			68			4
Cell adhesion			6			0.5
Cell Death	13				1	
Cell Growth & Maintenance	334			20		
Organization & Biogen		31			2	
Cell proliferation		21			1.5	
Transport		286			17	
Development	25			2		
Physiological Processes	1641			98		
Cell Growth & Maintenance		334			20	
Death		13			1	
Metabolism		1345			80	
Alcohol metabolism			61			4
Amine metabolism			83			5
Amino Acid metabolism			91			5.5
Aromatic cpd metabolism			51			3
Biosynthesis			498			30
Carbohydrate metabolism			154			9
Carbon utilization			19			1
Catabolism			160			9.5
Coenzyme & Prosthetic grp			38			2
Electron transport			162			9.5
Energy pathways			115			7
Heterocycle metabolism			19			1
Lipid metabolism			55			3
Nitrogen metabolism			8			0.5
Nucleic acid metabolism			174			10
One carbon cpd metabolism			10			0.5
Organic acid metabolism			102			6
Oxidative phosphorylation			13			1
Oxygen and ROS metabolism			26			1.5
Phosphorous metabolism			111			7
Pigment metabolism			7			0.5
Protein metabolism			536			32
Secondary metabolism			16			1
Sulfur metabolism			10			0.5
Vitamin metabolism			8			0.5
Photosynthesis		73			4	
Response to Endogenous Stimuli		16			1	
Response to External Stimuli		74			4	
Perception			14			1
Response to abiotic			22			1.5
Response to biotic			45			2.5
Response to Stress		61		3.5		
Response to DNA damage			16			1
Response to oxidative stress			23			1.5
Response to pest/pathogen			8			0.5
Response to water deprivation			6			0.5
Secretion		3				

**Table 3:** Gene ontology (GO) mappings: Cellular component 982 clusters

Categories and subcategories	Representation				% of Total			
Cell	963				98			
Intracellular		711				72		
Cell Cortex			6				0.5	
Chromosome			9				1	
Cytoplasm			563				57	
Cytoskeleton				34				3.5
Cytosol				27				2.5
Endoplasmic reticulum				29				3
Translation elongation cplx				6				0.5
Golgi apparatus				12				1
Mitochondrion				70				7
Plastid (Chloroplast)				122				12.5
Ribosome				224				23
Nucleus			109				11	
Ribonucleoprotein complex			233				24	
Ribosome				224				23
Membrane		368				37.5		
Endomembrane system			5				0.5	
Organelle Inner membrane			40				4	
Integral to membrane			159				16	
Unclassified				149				15
H+-transporting ATPase				10				1
Mitochondrial membrane			43				4	
Organelle Outer membrane			11				1	
Plasma membrane			5				0.5	
Extracellular	7				0.5			

**Table 4:** Gene ontology (GO) mappings: Molecular function 1992 clusters

Categories and subcategories	Representation			% of Total		
Antioxidant activity	6			0.5		
Binding	758			38		
Amino acid binding		6			0.5	
Carbohydrate binding		6			0.5	
Lipid binding		6			0.5	
Metal ion binding		161			8	
Calcium			43			2
Magnesium			18			1
Transition metal			78			4
Nucleic Acid binding		296			15	
DNA binding			125			6
Nuclease activity			15			1
RNA binding			58			3
Translation factor, nucleic acid			54			3
Nucleotide binding		335			17	
Purine nucleotide (adenyl/guanyl)			334			17
Protein binding		25			1.5	
Catalytic Activity	1162			58.5		
Helicase activity		25			1.5	
Hydrolase activity		374			19	
acting on acid anhydrides			108			5.5
acting on carbon-nitrogen (not peptide)			5			0.5
acting on ester bonds			71			3.5
acting on ether bonds			7			0.5
acting on glycosyl bonds			39			2
Peptidase activity			97			5
Isomerase activity		48			2.5	
Kinase activity		137			7	
Protein kinase activity			90			4.5
Lipase activity		55			3	
Lyase activity		93			4.5	
Oxireductase (Ored) activity		264			13.5	
disulfide Ored activity			17			1
Monooxygenase activity			28			1.5
Ored activity – CH-OH donors			55			3
Ored activity – acting on NADH/NADHP			13			0.5
Ored activity-acting on paired donors			13			0.5
Ored activity – peroxide acceptor			27			1.5
Ored activity – single donor			17			1
Ored activity – sulfur group donors			16			1
Oredactivity – CH-NH^2 ^group donors			9			0.5
Small protein conjugating enzyme activity		11			0.5	
Transferase activity		310			15.5	
transferring acyl groups			30			1.5
transferring alkyl or aryl groups			11			0.5
transferring glycosyl groups			35			2
transferring nitrogenous groups			12			0.5
transferring one–carbon groups			38			2
transferring phosphorous containing grps			154			7.5
Chaparone activity	45			2.5		
Defense protein activity	7			0.5		
Enzyme activator activity	6			0.5		
Unknown molecular function	50			2.5		
Nutrient reserve activity	8			0.5		
Signal Transducer activity	58			3		
Receptor activity		41			2	
Two-component response regulator		9			0.5	
Two-component response sensor molecule		18			1	
Structural Molecule activity	261			13		
Structural component of ribosome		239			12	
Transcription Regulator activity	41			2		
Transcription factor		27			1.5	
Two-component response regulator		9			0.5	
Translation Regulator activity	54			3		
Nucleic acid binding		54			3	
Transporter activity	286			14		
amine/polyamine transport		8			0.5	
carbohydrate transport		11			0.5	
Carrier activity		109			5.5	
electrochemical potential driven			29			1.5
primary active transporter			81			4
Channel/pore class transporter		25			1	
Electron transport		61			3	
Ion transport		57			3	
anion			5			0.5
cation			49			2.5
metal ion			16			1
Organic acid transport		11			0.5	
Protein transport		46			2.5	

Of the 2,203 clusters, 1673 (76%) map into the Biological Processes classification, 1641 (98%) of these fall into the Physiological Processes category and 409 (24.5%) cross-map into the Cellular Processes category (Table 2). Within physiological processes 80% of the clusters were associated with metabolism and 20% cell growth and maintenance (it is within this group that most of the overlap occurs with the Cellular Processes category). The distribution is not surprising for ESTs (clusters) derived from a tissue that is harvested at a time of metabolic upheaval such as rehydration and recovery from the desiccated state. In support of this notion are the almost identical distributions of ESTs observed for cDNA collections from protonemal tissues of the moss *Physcomitrella patens *following various hormonal treatments designed to illicit developmental perturbations and metabolic switching (Table 5, and from data reported by Nishiyama et al., 2003 [38]).

**Table 5:** Comparison of GO mappings for *Tortula ruralis *and *Phycomitrella patens*

**Relationship level**				**Gene ontology**	**ISC**	**P.pat**	**ISC**	**T.rur**	**T:P**
1					9981	%	2203	%	
**2**				**biological process**	**7119**	**71.33**	**1673**	**75.94**	**1.1**
	3			physiological processes	6901	69.14	1641	74.49	1.1
		4		metabolism	5601	56.12	1345	61.05	1.1
			5	biosynthesis	1672	16.75	498	22.61	1.3
			5	carbohydrate metabolism	642	6.43	154	6.99	1.1
			5	carbon utilization	44	0.44	19	0.86	2.0
			5	catabolism	670	6.71	160	7.26	1.1
			5	energy pathways	306	3.07	115	5.22	1.7
			5	oxidative phosphorylation	28	0.28	13	0.59	2.1
			5	oxygen and ROS metabolism	76	0.76	26	1.18	1.6
		4		photosynthesis	138	1.38	73	3.31	2.4
			5	photosynthesis, dark reaction	21	0.21	9	0.41	2.0
			5	photosynthesis, light reaction	56	0.56	41	1.86	3.3
		4		response to external stimulus	232	2.32	74	3.36	1.4
			5	perception of external stimulus	37	0.37	14	0.64	1.7
			5	response to abiotic stimulus	75	0.75	22	1.00	1.3
			5	response to biotic stimulus	136	1.36	45	2.04	1.5
		4		response to stress	231	2.31	61	2.77	1.2
			5	response to oxidative stress	57	0.57	23	1.04	1.8
			5	response to water deprivation	8	0.08	6	0.27	3.4
		4		cell growth and/or maintenance	1536	15.39	334	15.16	1.0
	3			cellular process	1845	18.49	409	18.57	1.0
		4		cell communication	310	3.11	75	3.40	1.1
			5	signal transduction	254	2.54	68	3.09	1.2
		4		cell growth and/or maintenance	1536	15.39	334	15.16	1.0
			5	cell organization and biogenesis	222	2.22	31	1.41	0.6
			5	cell proliferation	219	2.19	21	0.95	0.4
			5	transport	1131	11.33	286	12.98	1.1
	3			development	119	1.19	25	1.13	0.9
**2**				**cellular component**	**4290**	**42.98**	**982**	**44.58**	**1.0**
	3			cell	4137	41.45	963	43.71	1.1
			5	cell wall	73	0.73	7	0.32	0.4
		4		intracellular	2909	29.15	711	32.27	1.1
			5	cytoplasm	2041	20.45	563	25.56	1.2
			5	extrachromosomal DNA	37	0.37	15	0.68	1.8
			5	nucleus	815	8.17	109	4.95	0.6
			5	ribonucleoprotein complex	590	5.91	233	10.58	1.8
			5	thylakoid	116	1.16	46	2.09	1.8
		4		membrane	1628	16.31	368	16.70	1.0
			5	mitochondrial membrane	104	1.04	43	1.95	1.9
			5	inner membrane	100	1.00	40	1.82	1.8
			5	outer membrane	28	0.28	11	0.50	1.8
**2**				**molecular function**	**8755**	**87.72**	**1992**	**90.42**	**1.0**
	3			binding	3530	35.37	758	34.41	1.0
		4		nucleic acid binding	1389	13.92	296	13.44	1.0
			5	DNA binding	649	6.50	125	5.67	0.9
			5	nuclease activity	89	0.89	15	0.68	0.8
			5	RNA binding	330	3.31	58	2.63	0.8
			5	translation factor activity	176	1.76	54	2.45	1.4
		4		nucleotide binding	1478	14.81	335	15.21	1.0
		4		protein binding	221	2.21	25	1.13	0.5
		4		metal ion binding	616	6.17	161	7.31	1.2
	3			chaperone activity	202	2.02	45	2.04	1.0
		4		heat shock protein activity	57	0.57	17	0.77	1.4
	3			signal transducer activity	233	2.33	58	2.63	1.1
		4		two-component sensor molecule activity	38	0.38	18	0.82	2.2
		4		receptor activity	167	1.67	41	1.86	1.1
			5	transmembrane receptor activity	30	0.30	17	0.77	2.6
		4		structural constituent of ribosome	553	5.54	239	10.85	2.0
	3			transcription regulator activity	234	2.34	41	1.86	0.8
		4		transcription factor activity	150	1.50	27	1.23	0.8
	3			translation regulator activity	176	1.76	54	2.45	1.4
		4		translation factor, nucleic acid binding	176	1.76	54	2.45	1.4
			5	translation elongation factor activity	67	0.67	33	1.50	2.2
			5	translation initiation factor activity	97	0.97	20	0.91	0.9
	3			transporter activity	1115	11.17	286	12.98	1.2
		4		carbohydrate transporter activity	49	0.49	11	0.50	1.0
		4		carrier activity	414	4.15	109	4.95	1.2
		4		channel/pore class transporter activity	68	0.68	25	1.13	1.7
			5	alpha-type channel activity	59	0.59	23	1.04	1.8
	3			catalytic activity	5294	53.04	1162	52.75	1.0
		4		isomerase activity	183	1.83	48	2.18	1.2
			5	intramolecular isomerase activity	41	0.41	10	0.45	1.1
		4		kinase activity	735	7.36	137	6.22	0.8
		4		lyase activity	352	3.53	93	4.22	1.2
		4		oxidoreductase activity	960	9.62	264	11.98	1.2
		4		transferase activity	1557	15.60	310	14.07	0.9
		4		hydrolase activity	1764	17.67	374	16.98	1.0

The subcategory distributions within the Physiological and Cellular processes also seem to reflect the nature of the cellular disturbances that result from a desiccation-rehydration event. Processes involved in metabolite and ion transport within and between cells are represented by 17% of the clusters that map to Biological Processes, and almost 86% of those that map to the Cell Growth and Maintenance subcategory. Within the Metabolism subcategory of Physiological processes 40% (32% of total) of the clusters map to protein metabolism (synthesis) and 37% (30% of total) map to biosynthetic processes. The considerable representation within the aforementioned three subcategories is consistent with much of our biochemical and physiological evidence concerning the metabolic activity and emphasis in gametophytic cells during rehydration [15,24,25]. In particular, following rehydration the protein synthetic machinery is rapidly reconstituted, having been dismantled during drying, to direct the synthesis of pattern of proteins termed rehydrins that appear to be a crucial aspect of the desiccation tolerance mechanism of *Tortula ruralis *(as described above). The importance of the protein synthetic machinery and its re-establishment following rehydration is also highlighted by the preponderance of clusters associated with the Ribosomal subcategory of the Cellular Component Classification; 40% of the Cytoplasm category (23% overall).

Only 982 clusters (45% of the 2,203 that constitute the mapped population) map within the Cellular Component Classification and are presumably associated with structural functions (Table 3). Of these 982 clusters, almost all map to the Cell classification within which 72% map to Intracellular components and 38% to the Membrane category. Within these categories, representation is most significant in the ribosomal, integral membrane protein, and plastid subcategories. As discussed above the importance of protein synthesis during recovery from desiccation tolerance may explain the preponderance of clusters associated with ribosomal structural components as well as the number of clusters that are associated with the membrane and plastid subcategories. Ultrastructural studies of dried and rehydrated gametophytes clearly indicate that the inrush of water during rehydration disrupts membranes and causes a disorganization of the internal granal structures and swelling of the large chloroplasts of the *Tortula *leaf cells [36,39].

Of the clusters that can be mapped into GO hierarchies, 90% can be ascribed molecular functions (Table 4). Of the major categories, Binding activity (38%), Catalytic activity (58.5%), Structural Molecule activity (13%), and Transporter activity (14%) are best represented in the cluster collection. Metal ion, nucleic acid, and nucleotide binding are the most represented subcategories within the Binding activity category perhaps reflective of the need for biosynthetic and repair activity associated with rehydration of moss cells. Within the Catalytic activity category the majority of the clusters are associated with Hydrolase (19%), Transferase (15.5%), Oxireductase (13.5%), and Kinase (7%) activities. Almost half of the clusters associated with the Transferase activity subcategory map as transferring phosphate-containing groups. Each one of these subcategories represent catalytic activities that could be argued as important for a cell to recover from a major metabolic perturbation such as that seen during rehydration. The significant representation within the Kinase and phosphate transfer categories also suggests an active metabolic control "program" occurs when the desiccated cells receive water and attempt to recover from the damage. It is also intriguing that there is a significant representation within the Transporter subcategory as little is known of this group within the context of desiccation tolerance in bryophytes, although solute (osmolytes) and sugar transport in and out of the vacuoles of desiccation tolerant Angiosperms [3] do occur during desiccation and rehydration.

Of particular interest to this study are clusters that represent gene expression control factors both at the transcriptional (41 clusters) and translational level (54 clusters). These clusters, along with those that represent biochemical control mechanisms for signaling and gene expression at the protein level, such as kinases (90 clusters) and phosphate-transfer activities (154 clusters), may represent critical elements in the activation and execution of the cellular recovery processes necessary for the mechanism of desiccation tolerance exhibited by *Tortula ruralis*. This is of importance because of the key position of bryophytes in the evolution of desiccation tolerance in plants. Our main hypothesis is that an elucidation of the signaling and activation pathways for the rehydration response in this assumed primitive tolerance mechanism could have major implications for the study of stress tolerance mechanisms in all plants and thus these clusters represent important targets for further study at the molecular and biochemical levels.

### GO based comparison with *Physcomitrella patens *EST collections

The representation of the *Tortula *rehydration clusters throughout the GO mapping system is indicative of the emphases on, but not expression levels of, particular cellular activities represented in the moss gametophytes during this period of a wet and dry cycle. In an attempt to assess if the accents on particular cellular activities indicated for rehydrated gametophytes are characteristic of the rehydration induced metabolic state or are simply indicative of processes associated with normally active bryophyte cells, we compared the representation of the *Tortula *rehydration clusters within the GO categories with similar "clusters" from *Physcomitrella patens*, the only other bryophyte that has similar genomic level information. The majority of the *Physcomitrella *"clusters" are derived from a large EST collection representing transcripts from both untreated and hormone induced (to switch developmental pathways) cells of protonemal cultures. The *Physcomitrella *ESTs are described by Nishiyama et al., [38] and were obtained from Physcobase  as assembled contigs (assembled in an identical fashion to what we designate as clusters). In total 22,885 *Physcomitrella *contigs, derived from 102553 ESTs obtained from Physcobase and Genbank, were subjected to a BLASTx search, as described for the *Tortula *clusters using HT-GO-GAT. Of the 22,885 contigs 9,981 (43.6%) represent genes that contain conserved protein domains that have known biochemical and physiological functions in other organisms and map to the GO hierarchy.

There are two caveats for this comparison; 1) differences in representation may simply reflect species differences in the emphasis on individual classes of cellular activities between *Tortula *and *Physcomitrella*, or 2) differences in representation may reflect differences in the emphasis on individual classes of cellular activities between mature gametophytes (*Tortula*) and protonema (*Physcomitrella*). Until a comparison of GO mapping distributions can be made directly between clusters derived from an EST collection from hydrated control gametophytes from *Tortula *with those from the rehydration collection these caveats remain important. Nevertheless, even with these difficulties and limitations the comparison is still useful for developing new hypotheses as to what cellular processes might be important in the recovery of moss cells from desiccation.

In general the distribution of the *Tortula *clusters within the GO mappings are similar if not identical to the distribution of the Phycomitrella contigs gleaned from Physcobase. This can be seen especially at the GO map 2 and 3 relationship-level categories (Table 1 and supplemental material) using the multi-species GO browser (DrZOOview2.0 ). However, there are several notable differences and in general the differences are consistent with what has been determined, in earlier studies, to be important in the recovery of the moss from desiccation following rehydration [4,23] and what is known about plant responses to abiotic stress in general [8,13]. The differences evident in the comparison are presented in Table 5, where the extent of the similarity in the distribution of the clusters are expressed as the ratio of representation for *Tortula *to representation for *Physcomitrella *(T:P). In the Biological category the most striking differences in GO representation of *Tortula *clusters occurs in categories where the percentage representations are relatively low but the numbers of clusters are substantial. In the level 4 relationship, Photosynthesis, the representation for *Tortula *is 3.31% compared to 1.38% for *Physcomitrella*, a 2.4 fold difference, the majority of which is accounted for by the difference in the representation levels for the light reaction category. The rapid recovery of photosynthesis is critical for the recovery of bryophyte cells, particularly in regards to the production of energy and reducing power for the metabolic activity associated with repair and reconstitution of the gametophytic cells. Chloroplast structure in *Tortula *is severely disrupted, especially if desiccation occurred rapidly, in the first few hours following rehydration [36,40] but recovers quickly. The difference in representation in this category is consistent with these observations and thus may reflect the greater need for a supply of a diversity of photosynthetic components in rehydrated *Tortula *than in *Physcomitrella* cells that have not experienced a disruption in the photosynthetic apparatus. Similar inferences can be made concerning the differences in representation observed for carbon utilization (T:P of 2.0) and oxidative phosphorylation (T:P of 2.1) for *Tortula *in that mitochondrial activity and integrity are also compromised during rehydration [25,36]. Other differences in representation between *Tortula *and *Physcomitrella *mappings relate to a higher representation for *Tortula *in the categories that relate to responses to external stimuli and responses to stress both of which would seem consistent with the emphasis that cellular activity for *Tortula *would have in comparison to unstressed *Physcomitrella *cells. In particular the increased representation within the response to oxidative stress is of note, as an elevated protection of cellular integrity from the damaging reactive oxygen species (ROS) typically associated with a desiccation rehydration event is a distinctive component of desiccation tolerant bryophytes when compared to their desiccation sensitive relatives such as *Physcomitrella *[41].

The above observations concerning the more extensive representation of clusters within the GO mappings related to organelle function in *Tortula *are mirrored in the Cellular Component Level 2 classification mappings. In the Cellular Component classification *Tortula *exhibits an almost two fold difference in representation within categories associated with either the chloroplast or mitochondria, such as Extrachromsomal DNA (T:P of 1.8), Thylakoid (T:P of 1.8), Mitochondrial membrane (T:P of 1.9), and both Inner and Outer membranes (T:P of 1.8). In this case the categories are generally related to genes representing membrane components and since it is the organelle membranes that exhibit the majority of the damage during desiccation and rehydration it is consistent that these categories would be better represented in the *Tortula *cluster mappings than those for *Physcomitrella*. In addition to the organellar related classifications the *Tortula *clusters also exhibit a higher representation within the Ribonucleoprotein complex category (T:P of 1.8) which in all likelihood reflects an emphasis on ribosomal components since a similar difference in representation is seen in the Structural Constituent of the Ribosome category (T:P of 2.0) within the Molecular Function Level 2 classification. Again such differences in representation in the comparison between *Tortula *and *Physcomitrella *GO mappings are consistent with our previous studies on the responses of *Tortula *gametophytes to desiccation and rehydration and comparisons to non-stressed bryophyte tissues. Protein synthesis is critical to the recovery of *Tortula *cells following a desiccation event [11,13,15] not only for the synthesis of proteins damaged by the stress of desiccation but also directing the response to the stress at the level of gene expression [26,29]. Early studies determined that the speed at which desiccation occurred has a marked effect on both the rate of recovery of protein synthesis and the rate at which either new ribosomes are formed or pre-existing ones are repaired [42,43], rapid desiccation results in a more prolonged recovery of normal protein synthetic levels and also slows the reconstitution of ribosomes upon rehydration. Since the *Tortula *clusters are derived from ESTs of rehydrated moss that was dried rapidly, the greater representation in ribosome related GO mappings for this collection compared to the *Physcomitrella *clusters, that represent transcripts from cells where presumably normal ribosomal turnover and synthetic rates are prevalent, is consistent with the biological state of the *Tortula *cells. The emphasis on protein synthesis in rehydrated *Tortula *cells compared to those of *Physcomitrella *is also evident in the comparison of representations within the Translation regulator, Translation factor, and Translation elongation factor activity GO mappings.

Other differences seen in the Molecular function classification, such as the greater representation within the *Tortula *collection of clusters involved in Two-component Sensor or Channel/pore class Transporter activity designated mappings, offer novel possibilities for investigation into rehydration metabolism that have not been indicated as important until now. The individual identity of the clusters that map to these categories should offer possible hypotheses that can be tested in our future research.

### Functional classification based on KEGG analysis

An alternative classification of clusters, based on biochemical function, involves the use of HT-GO-GAT to assign clusters to individual Kyoto Encyclopedia of Genes and Genomes (KEGG ) metabolic pathways. Of the 2203 clusters that map within the GO hierarchy only 642 clusters had assigned EC numbers generating 325 unique mappings (see additional file 1: KEGG biochemical pathway mappings for Tortula rehydration clusters). The paucity of EC assignments limits this aspect of the analysis but the mapping of clusters to the KEGG metabolic pathways still presents some useful perspectives on the metabolic emphasis of the rehydrated gametophytic cells. Eighty-six of the 123 pathways contained within the Metabolism category (metabolic pathways), were represented by 84.6% of the 642 *Tortula *clusters. The KEGG metabolic pathways that are well represented by *Tortula *clusters are Carbohydrate Metabolism (83 enzymes represented), Amino Acid Metabolism (72 enzymes), Energy Metabolism (40 enzymes), Lipid Metabolism (27 enzymes), and Metabolism of Cofactors (24 enzymes). All of these pathways have been previously associated with the cellular recovery processes associated with rehydrated moss gametophytes [14,15]. Within the metabolic activities not represented by the *Tortula *clusters only the lack of ascorbate metabolizing enzymes appears unusual as ascorbate has been well documented as an important metabolite in the protection of moss gametophytes from oxidative damage during stress. This however appears to be the result of a limitation in the assignment of EC numbers since several *Tortula *clusters show significant similarities to enzymes involved in ascorbate metabolism in the original BLASTx search used to generate the GO mappings. The limitation of the KEGG based classification can also been seen in the poor representation of *Tortula *clusters in the other KEGG pathways (Genetic Information Processing, Environmental Information processing, and Cellular Processing) which is surprising given the number of clusters that were identified in the BLASTx search and in the GO databases as being contained within these classifications. As an example, none of the 239 clusters that map in the GO hierarchy as structural components of the ribosome (Table 4) or any of the clusters that mapped to transcriptional and translational components were contained in the corresponding KEGG pathways. Thus although useful information can be gleaned from the KEGG classification system and metabolic pathway mappings, especially in practical terms for functional studies using individual clusters, it has some major limitations for drawing any broad based hypotheses from the representation of *Tortula *clusters within each pathway.

### ORF based assessment of *Tortula *clusters

**Figure 2:**
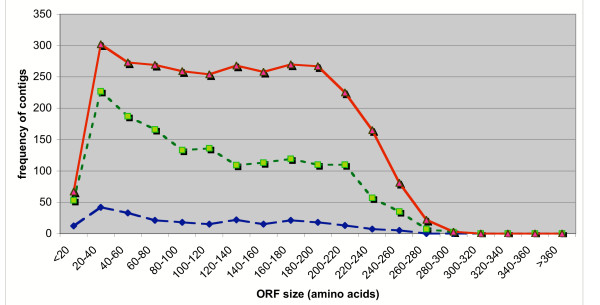
**Distribution of clusters by size of longest ORF (by number of amino acids)**. **Solid line**, clusters that have significant similarity with a known sequence in the database by BLASTX. **Dotted Line**, clusters that showed weak similarity with a known sequence in the database. **Dashed Line**, clusters without similarity to known sequences in the database.

Of the 5,563 consensus cluster sequences used in the BLASTx search of our database (see above) 40.3% failed to exhibit sufficient similarity (did not meet set criteria, see above) with known sequences to allow for an accurate annotation of the cluster. It is possible that these contigs, rather than containing novel amino-acid coding regions, contain mainly 3' or 5' untranslated regions (UTRs) or coding regions that are so short as to render them incapable of generating a significant similarity score. In order to investigate this possibility we examined the three classes of contigs, those with significant similarity scores; "good hits", those that generated poor similarity scores; "false hits", and those that failed to generate any scored similarity; "no hits", to determine the longest open reading frame (ORF). We limited the ORF determination to those clusters that contain an AUG codon in the 5' to 3' direction of the clone in any one of the three possible reading frames (the cDNAs were directionally cloned). The results of this analysis are shown in Figure 2. Of the 5,563 clusters generated in the study, 4,789 generated ORFs under the limitations imposed by the analysis. Of these 2,983 were classified as "good hits", 1,564 as "false hits" and 242 as "no hits". Those clusters that are classified as "good hits" exhibit ORFs that are in general evenly distributed from 20–40 amino acids long to 220–240 amino acids long. The clusters that are classified as "false hits" from the BLASTx search do have a relatively larger proportion of shorter ORFs, in the 20–40 and 40–60 amino acid range but also a substantial proportion that are much longer. The distribution of ORFs in this category ('false hits") does not appear to be sufficiently skewed from that for the "good hit" clusters to render them incapable of generating similarity scores in the BLASTx search. This would suggest that these clusters do contain novel amino acid sequences that are not represented in the public protein databases by sequences sufficiently similar to generate significant HSP Bit scores. In addition, the distribution of ORFs in the "no hit" cluster category are distributed in a similar manner to those of the "good hit" classification and so are also likely to represent clusters encoding proteins with novel amino acid sequences. The clusters contained within the "false hit" and "no hit" categories are of particular interest in our search for novel genes and pathways that are associated with the ability of certain plants to acquire vegetative desiccation tolerance.

### Conserved gene comparison to *Physcomitrella *and *Arabidopsis*

In this analysis the *Physcomitrella *and *Arabidopsis *databases were independently used in a BLASTx search using the 5,563 *Tortula *clusters as query sequences. Even though we used the entire 5,563 cluster sequences as individual queries only 3,321 actually represent sequences that could be annotated using the criteria for similarity discussed previously. When the *Tortula *clusters were used as queries against the *Physcomitrella *database, 5,554 generated HSP Bit scores however only 282 exhibited a level of conservation of sequence that passed the criteria established for annotation of our *Tortula *clusters (HSP Bits score above 70 and or E values of 10^-7 ^or less, see above). Against the *Arabidopsis *database only 927 *Tortula *clusters generated HSP Bit scores but 612 had a level of conservation sufficient to be considered reliable matches to *Arabidopsis *genes. These observations are difficult to rationalize and may simply reflect the inequality of the target databases. The *Physcomitrella *database, which is generated from a relatively small EST collection, is much smaller than that for *Arabidopsis*, which is gleaned from the full genomic sequence. This may explain why although most *Tortula *clusters generated HSP Bit scores only a relative few were capable of producing scores sufficiently high enough, and with low enough error probabilities, for confident assignment of co-identity with a *Physcomitrella *contig. Obviously *Tortula ruralis *is more closely related to *Physcomitrella patens*, as they are both bryophytes, than to *Arabidopsis thaliana *but the phylogenetic distance between the two bryophytes is still substantial, which may also help explain the paucity of high value matches and the large number of low value hits. The *Arabidopsis *database is much more comprehensive and would be expected to generate more significant matches as observed and perhaps because of the evolutionary distance between the two plants more queries that do not generate an HSP.

The twenty gene products that exhibit the highest level of conservation for both comparisons, *Physcomitrella *(E values of 0 to -39) and *Arabidopsis *(E values of 0 to -55) are presented in Table 6. Several common highly conserved genes are present in both comparisons, including genes involved in cell structure (tubulin and actin), protein synthesis (ribosomal proteins and elongation factors), protein-turnover (polyubiquitins), stress proteins (heat shock), chromosomal proteins (histones), signal transduction (ADP-ribosylation factor), and binding proteins (calmodulin). Interestingly there are differences between the two lists, which may reflect the relative phylogenetic distances between the three species. The three most conserved proteins between *Tortula *and *Physcomitrella *did not register as highly conserved between *Tortula *and *Arabidopsis*, the most conserved protein between *Tortula *and *Physcomitrella*, the rRNA intron-encoded homing endonuclease, did not generate a match at all with an *Arabidopsis *counterpart. Interestingly, the most conserved photosynthesis related protein between *Tortula *and *Physcomitrella *is a chlorophyll a/b-binding protein which generates a HSP Bit score of 205 and E value of 3.0E^-53^, the *Arabidopsis *counterpart generates a HSP Bit score of 82 and E value of 8.9E-15. In this case the level of conservation of this protein within the comparisons appears to reflect the phylogenetic relationships that exist between the three plants. However, the most conserved photosynthesis related protein between *Tortula *and *Arabidopsis *is a photosystem I P700 apoprotein A2 which generates a HSP Bit score of 1120 and E value of 0, the *Physcomitrella *counterpart generates a HSP Bit score of 34 and E value of 1.25E^-1^, which appears contrary to what is expected at what is seen for the chlorophyll a/b-binding protein. This type of relationship is also seen for the Ribulose 1 protein (Cluster 2170) that is conserved between *Tortula *and *Arabidopsis *but only to a low degree with the *Physcomitrella *counterpart. If such relationships can be substantiated with further study and complete sequences (both the *Tortula *and *Physcomitrella *clusters are based on ESTs) then some interesting evolutionary questions can be raised concerning both gene function and selection pressures on the individual plants and metabolic processes.

**Table 6:** The twenty most conserved *Tortula *gene clusters between Physcomitrella and Arabidopsis

***Tortula *Cluster**	**Physcomitrella Identity**	**Bit Score**	**e-value**
Contig_2145	PPCContig_497 Q9AY32 (Q9AY32) RRNA intron-encoded homing endonuclease	813	0.0E+00
Contig_1352	PPCContig_406 Q9AVH2 (Q9AVH2) Putative senescence-associated protein (Fra	630	0.0E+00
Contig_3546	PPCContig_1008 O04892 (O04892) Cytochrome P450 like_TBP (EC 1.14.14.1)	448	1.8E-126
Contig_602	PPCContig_359 polyubiquitin UBQ10/SEN3	413	1.2E-115
Contig_480	PPCContig_12 Q9SPA1 (Q9SPA1) Elongation factor-1 alpha 3	306	1.4E-83
Contig_966	PPCContig_1903 EF1A_VICFA (O24534) Elongation factor 1-alpha (EF-1-alpha)	284	5.8E-77
Contig_1126	PPCContig_2103 Q41067 (Q41067) Polyubiquitin	250	5.9E-67
Contig_1901	PPCContig_1954 Q8H932 (Q8H932) Alpha tubulin	241	7.4E-64
Contig_1538	PPCContig_4431 heat shock protein hsc70-1 (hsp70-1) (hsc70.1)	233	1.6E-61
Contig_1444	PPCContig_49 ADP-ribosylation factor 1 (ARF1), putative	208	4.6E-54
Contig_919	PPCContig_1049 Q9SXW8 (Q9SXW8) Chlorophyll a/b-binding protein	205	3.0E-53
Contig_1381	PPCContig_2023 Q8S173 (Q8S173) Putative 60S ribosomal protein L37a	197	5.3E-51
Contig_5058	PPCContig_951 Q9SWW8 (Q9SWW8) Actin (Fragment)	195	3.3E-50
Contig_1676	PPCContig_2347 calmodulin	193	8.7E-50
Contig_1366	PPCContig_846 Q84NX8 (Q84NX8) Putative ribosomal protein L19	174	8.0E-44
Contig_2369	PPCContig_2689 O04664 (O04664) Small RAS-like GTP-binding protein (AT5G551 173		9.9E-44
Contig_1934	PPCContig_4083 Q9SJB9 (Q9SJB9) Putative translation initiation factor eIF-	171	3.3E-43
Contig_3793	PPCContig_3975 Q9LFN6 (Q9LFN6) DEAD box RNA helicase RH15	169	1.9E-42
Contig_1721	PPCContig_2679 H33_ARATH (P59169) Histone H3.3	165	2.1E-41
Contig_3462	PPCContig_970 Q43821 (Q43821) Ubiquitin conjugating enzyme (EC 6.3.2.19)	160	1.2E-39
***Tortula *Cluster**	***Arabidopsis *Identity**	**Bit Score**	**e-value**
Contig_3469	ATCG00340 (PSAB) photosystem I P700 apoprotein A2	1120	0.0E+00
Contig_882	AT1G07930. elongation factor 1-alpha (EF-1-alpha)	666	0.0E+00
Contig_602	AT4G05320.4 polyubiquitin UBQ10/SEN3	617	1.0E-175
Contig_2170	ATCG00490 (RBCL) riblose 1	435	5.0E-121
Contig_1984	AT5G02960.1 40S ribosomal protein S23 (RPS23B)	431	5.2E-120
Contig_1538	AT5G02500.1 heat shock protein hsc70-1 (hsp70-1) (hsc70.1)	366	3.2E-100
Contig_1124	AT4G05050.1 polyubiquitin UBQ11	341	5.8E-93
Contig_1444	AT1G23490.1 ADP-ribosylation factor 1 (ARF1), putative	307	1.2E-82
Contig_1901	AT1G50010.1 tubulin alpha-2/alpha-4 chain (TUA2)	303	3.7E-81
Contig_52	AT3G12580.1 heat shock protein hsp70	291	1.3E-77
Contig_1060	AT4G38510.1 probable H+-transporting ATPase	285	4.1E-76
Contig_1125	AT4G02890.2 polyubiquitin (UBQ14)	274	8.3E-73
Contig_3069	AT4G40040.1 histone H3.2	270	1.4E-71
Contig_1935	AT5G08290.1 YLS8, Dim1 homolog	264	1.1E-69
Contig_2698	AT5G45775.2 60S ribosomal protein L11 (RPL11D)	260	1.2E-68
Contig_5058	AT5G09810.1 ACTIN 2/7 (sp|P53492)	258	6.8E-68
Contig_1676	AT3G43810.1 calmodulin	252	2.8E-66
Contig_3867	AT3G05530.1 26S proteasome AAA-ATPase subunit RPT5a	232	3.4E-60
Contig_347	AT3G11940.2 40S ribosomal protein S5 (RPS5B)	230	2.2E-59
Contig_1052	AT2G29550.1 tubulin beta-7 chain (TUB7)	218	1.3E-55

## Conclusions

Bryophytes have an important and underestimated place in the study of plant responses to water deficits, in particular desiccation tolerance. Bryophytes occupy what we believe to be one of the most primitive states, along with algae, in the evolution of desiccation tolerance and represent, in all probability, the stage in the emergence of plants from a fresh water environment to occupy the various niches available on dry land [13]. Unfortunately, it is only with the advent of the development of *Physcomitrella patens *as a model plant for molecular genetic studies, fired by its particular ability to perform efficient and homologous recombination in vitro, that bryophyte genomics has become a topic of some interest. Physcomitrella is rapidly becoming the model of choice for developmental and transgenic studies [44] because of its ease of manipulation and indeed there are plans in place to sequence its genome [45]. *Tortula ruralis *on the other hand has long been established as an attractive model for the analysis of environmental stress tolerance, in particular desiccation tolerance, It has been a very useful model in assessing structural, physiological, biochemical and genetic (gene expression) aspects of severe dehydration of plant cells and mechanisms by which primitive plants respond to and survive protoplasmic water loss [15,25,46]. The progression of the *Tortula *model into genomics is a critical aspect in the development, along with a transformation system and assessment of its ability for efficient homologous recombination, into a more useful and manipulable model for understanding desiccation tolerance and the nature of extremophiles. In addition to the importance of *Tortula *for stress biology, the establishment of a second and contrasting bryophyte model, especially with regards to genomics, is essential for the validation and usefulness of the information gained from the analysis of the *Physcomitrella *genome and the general principles gleaned from its use as a plant model. It is to these ends that we have initiated this study into the transcriptome of *Tortula *gametophytes as they respond to a major stress event, in this case a combination of desiccation and rehydration.

The rehydration transcriptome of *Tortula*, as defined by the clusters presented herein, is remarkably consistent with what we know about the desiccation response for this bryophyte and its metabolic activity during the first two hours following rehydration. The GO mapping of the *Tortula *clusters enabled a broad look at what cellular activities appear to be emphasized in the rehydrated gametophytes and in agreement with our previous biochemical analyses highlighted the prominence of the protein synthetic machinery, both in structure and control, membrane structure and metabolism, and the need to reestablish plastid integrity. These observations were bolstered by the comparative GO analysis using the extensive EST collection generated for the desiccation sensitive moss *Physcomitrella patens*. The GO analysis has also provided fuel for new investigations and hypotheses into the role of other cellular processes, such as membrane transport, phosphorylation and signal transduction, in the mechanisms that enable desiccation tolerance in plants. Signal transduction is especially intriguing with regards to desiccation tolerance in this bryophyte as it appears to rely on alterations in translational control to effect a response to desiccation in contrast to the well characterized transcriptional responses, and associated signaling pathways, associated with abiotic stress in the Angiosperms [5,7]. In addition to the functional based analyses, the simple abundance estimates has also correlated well with previous work and has given further credence to the notion that LEA proteins may also play a role in maintaining cellular integrity when water is reintroduced into desiccated plant tissues. The strong correlation between what is known about the mechanism for desiccation tolerance employed by *Tortula ruralis *and what can be inferred from the analyses of the *Tortula *rehydration EST collection gives a measure of confidence not only in the value of a bioinformatics approach to gain a view of a particular transcriptome but also in the basis for new hypotheses and research directions that are generated from them.

Although the type of analyses presented here are extremely useful in assessing the types of transcripts present in a particular tissue at a particular time and in response to some perturbation, either external or internal in origin, and generating hypotheses concerning the functional aspects of the transcriptome they are intrinsically correlative in nature. In order to gain a more direct picture of the transcriptome and more importantly, with regards to functional assessments, the "translatome", the bioinformatics must be linked to a detailed expression profile of the transcripts represented within the ESTs generated to investigate the particular biological response or mechanism of interest. To this end the clusters described in this report form the basis of a *Tortula *gametophyte microarray designed for the profiling of both the extant transcriptome and the associated translatome and their response to desiccation and rehydration under various conditions. The array and the experimental design of the expression profiling will allow us to generate a more accurate assessment of both transcript levels and transcript recruitment into the protein synthetic machinery during, and recovery from, desiccation in *Tortula ruralis*. In combination with the bioinformatics analyses presented here, the expression profiling will allow us to generate a more complete picture of the cellular response of a tolerant plant species, an extremophile, to an extreme abiotic stress event.

## Methods

### Source material

*Tortula ruralis *([Hedw.] Gaertn, Meyer and Scherb), also classified as *Syntrichia ruralis*, gemetophytes were collected, harvested, and stored as described previously [27]. For experimental purposes, gametophyte tissue was hydrated for 48 h to fully recover from dried storage and trimmed to remove stem material. Rapid-dried moss was prepared by placing the cropped gametophytes in a closed atmosphere of 0% relative humidity (RH) on 3-mm filter paper over activated silica gel in a Petri dish. This drying regime resulted in the attainment of the air-dried state within 30 min. The gametophytes remained in this atmosphere overnight to ensure desiccation and prior to library construction were rehydrated for 2 h in deionized water at 18°C in the light.

### Library construction

Total RNA was isolated from the rehydrated gametophytes by a series of phenol extractions as described by Lane and Tumiatis Kennedy [47]. PolyA RNA was isolated from the total RNA fraction by oligo-(dT) chromatography [48], using DynaBeads oligo-(dT25) (Dynal, Inc., Lake Success, NY, USA), through two rounds of selection according to the manufacturer's instruction. The purified polyA fraction was used as a template for double-stranded cDNA synthesis using the Superscript Plasmid System (Invitrogen Life Sciences, Carlsbad, CA, USA). The resultant cDNA population was cloned into the pSPORT1 vector, according to manufacturer's instructions, to construct the unidirectional rehydration cDNA library. Small scale sequencing of 384 random clones confirmed the directional aspect of the inserts, the plant nature of the source cDNAs, and the frequency of positive clones. The average insert length for the library was assessed at 1.2 Kb. A subset of 10, 368 randomly picked positive clones (white in a blue-white X-Gal/IPTG based screen) were transferred to individual wells in 384 well plates containing suitable growth medium for storage, replication, and sequencing.

### Sequence analysis

High throughput sequencing of the inserts contained in the 10,368 individual clones was performed using "rolling circle amplification" of the individual plasmids to generate suitable sequencing templates at the Joint Genome Institute, Walnut Creek, CA, U.S.A. Clones were sequenced using primers specific for vector sequence upstream of the multiple cloning site and at the 5' end of the cDNA insert. The sequences were delivered as primary binary files (raw trace files), which were then processed through our sequencing, pipeline as described below.

### Sequence preparation

Trace files were entered into SeqMan II and quality screening performed with medium stringency corresponding to a phred threshold value of 12. Vector searching was performed using the pSPORT™ vector both in forward and reverse orientations with minimum match length of 7, connect distance of 3, Maximum register shift of 10, Minimum NW percent match of 90, gap weight of 0 and length weight of 2. Contaminant screening was performed with minimum match of 25. Following the quality screening the sequences were exported as a single FASTA file and polyA tails were removed using a custom PERL script. Several hundred sequences that contained internal polyA stretches were manually identified. These sequences were manually edited to remove polyA and trailing sequences.

### Contigs

Edited sequences were then entered into SeqMan for assembly into contigs. Assembly for contigs was performed with match size of 50, minimum match percentage of 97, minimum sequence length of 100.

### Clusters

Consensus contig sequences were exported from SeqMan as individual files and entered into a separate SeqMan project and reassembled using the same parameters used for contigs but with a minimum match percentage of 90. These were considered clusters.

### EST submission

EST submission to GenBank was performed using the USDA-ARS Livestock Issues Research Unit's (LIRU) High Throughput-Gene Ontology-Genome Annotation Toolkit (HT-GO-GAT). HT-GO-GAT can be obtained from the LIRU website at . A total of 9159 EST sequences were submitted to GenBank and assigned accession numbers CN200321-CN209479

### Functional genetics

Functional annotations, Enzyme commission numbers, and Kyoto Encyclopedia of Genes and Genomes (KEGG) pathway assignments were assigned to cluster sequences using HT-GO-GAT. Consensus sequences derived from Clusters were entered into the software that utilizes custom BLASTx, RPS-BLAST, and relational mySQL databases to identify potential functional assignments based upon sequence and functional domain similarity matching. The software was set to identify high stringency matches and the resulting data manually curated using the high throughput results viewer interface of HT-GO-GAT. HT-GO-GAT produces various reports related to given datasets including ORF analysis, KEGG pathway reports and visualization, and also exports gene ontology association files. These association files were imported into a mySQL database and visualized using the USDA-ARS-LIRU's DrZooView2.0 GO database browser .

## Authors' contributions

MJO conceived of the study, generated the libraries and ESTs, annotated the EST database, performed much of the analyses, interpreted the results, and drafted the manuscript. SED designed the bioinformatics software and database, performed the sequence alignments, performed the ORF analysis, and generated the Bryobase website. JZ coded the HT-GO-GAT software and created the mySQL database. SAM participated in the design of the bioinformatics and design of the sequence analysis. PRP participated in the design of the database and sequence analysis, and participated in the annotation of the individual EST sequences.

## Supplementary Material

Additional File 1 **KEGG biochemical pathway mappings for *Tortula *rehydration clusters**. An alternative functional classification of clusters based on established biochemical activities of gene products. The classification is achieved by the use of HT-GO-GAT to assign clusters to individual Kyoto Encyclopedia of Genes and Genomes (KEGG ) metabolic pathways. This file contains the tabulated results of this classification. Click here for file
