# Supplementary material for: The rehydration transcriptome of the desiccation-tolerant bryophyte Tortula ruralis: transcript classification and analysis
Source: BMC Genomics. 2004 Nov 16;5:89. doi: 10.1186/1471-2164-5-89 (PMC535811; doi:10.1186/1471-2164-5-89)
Supplement: Additional File 1 — KEGG biochemical pathway mappings for Tortula rehydration clusters. An alternative functional classification of clusters based on established biochemical activities of gene products. The classification is achieved by the use of HT-GO-GAT to assign clusters to individual Kyoto Encyclopedia of Genes and Genomes (KEGG ) metabolic pathways. This file contains the tabulated results of this classification. [file 1471-2164-5-89-S1.doc]

| **KEGG biochemical pathway mappings for *Tortula* rehydration clusters** | | | |
| --- | --- | --- | --- |
| **Category** | **Pathway** | **#clusters** | **#of enzymes** |
|  | **Metabolism** |  |  |
| 1.1 Carbohydrate Metabolism |  |  |  |
| 1.1.1 | Glycolysis / Gluconeogenesis | 22 | 12 |
| 1.1.2 | Citrate cycle (TCA cycle) | 10 | 6 |
| 1.1.3 | Pentose phosphate pathway | 10 | 7 |
| 1.4 | Nucleotide sugars metabolism | 2 | 2 |
| 1.7.1 | Starch and sucrose metabolism | 16 | 9 |
| 1.7.5 | Aminosugars metabolism | 2 | 2 |
| 1.1.4 | Inositol metabolism | 10 | 3 |
| 1.1.5 | Pentose and glucuronate interconversions | 5 | 3 |
| 1.1.6 | Fructose and mannose metabolism | 10 | 8 |
| 1.1.7 | Galactose metabolism | 7 | 5 |
| 1.1.9 | Pyruvate metabolism | 19 | 9 |
| 1.1.10 | Glyoxylate and dicarboxylate metabolism | 8 | 5 |
| 1.1.11 | Propanoate metabolism | 7 | 6 |
| 1.1.12 | Butanoate metabolism | 10 | 5 |
| 1.1.1 | C5-Branched dibasic acid metabolism | 1 | 1 |
| 1.2 Energy Metabolism |  |  |  |
| 1.2.1 | Oxidative phosphorylation | 12 | 7 |
| 1.2.2 | ATP synthesis | 18 | 1 |
| 1.2.3 | Photosynthesis | 1 | 1 |
| 1.2.4 | Methane metabolism | 17 | 5 |
| 1.2.5 | Carbon fixation | 23 | 13 |
| 1.2.6 | Reductive carboxylate cycle (CO2 fixation) | 5 | 3 |
| 1.2.7 | Nitrogen metabolism | 17 | 8 |
| 1.2.8 | Sulfur metabolism | 2 | 2 |
| 1.3 Lipid Metabolism |  |  |  |
| 1.3.1 | Fatty acid biosynthesis (path 1) | 1 | 1 |
| 1.3.2 | Fatty acid biosynthesis (path 2) | 2 | 2 |
| 1.3.3 | Fatty acid metabolism | 6 | 5 |
| 1.3.4 | Synthesis and degradation of ketone bodies | 1 | 1 |
| 1.3.5 | Sterol biosynthesis | 4 | 4 |
| 1.3.6 | Bile acid biosynthesis | 2 | 2 |
| 1.8.1 | Glycerolipid metabolism | 13 | 8 |
| 1.8.3 | Inositol phosphate metabolism | 1 | 1 |
| 1.8.5 | Phospholipid degradation | 6 | 2 |
| 1.8.11 | Prostaglandin and leukotriene metabolism | 1 | 1 |
| 1.4 Nucleotide Mtabolism |  |  |  |
| 1.4.1 | Purine metabolism | 11 | 8 |
| 1.4.2 | Pyrimidine metabolism | 5 | 3 |
| 1.5 Amino Acid metabolism |  |  |  |
| 1.5.2 | Glutamate metabolism | 17 | 10 |
| 1.5.3 | Alanine and aspartate metabolism | 10 | 5 |
| 1.5.4 | Glycine, serine and threonine metabolism | 10 | 8 |
| 1.5.5 | Methionine metabolism | 9 | 5 |
| 1.5.6 | Cysteine metabolism | 3 | 2 |
| 1.5.7 | Valine, leucine and isoleucine degradation | 3 | 3 |
| 1.5.8 | Valine, leucine and isoleucine biosynthesis | 6 | 3 |
| 1.5.9 | Lysine biosynthesis | 1 | 1 |
| 1.5.10 | Lysine degradation | 4 | 2 |
| 1.5.11 | Arginine and proline metabolism | 6 | 4 |
| 1.5.12 | Histidine metabolism | 5 | 5 |
| 1.5.13 | Tyrosine metabolism | 14 | 7 |
| 1.5.14 | Phenylalanine metabolism | 13 | 5 |
| 1.5.15 | Tryptophan metabolism | 12 | 5 |
| 1.5.16 | Phenylalanine, tyr and tryp biosynthesis | 11 | 7 |
| 1.6 Metab of other Amino Acids |  |  |  |
| 1.6.1 | beta-Alanine metabolism | 5 | 2 |
| 1.6.2 | Taurine and hypotaurine metabolism | 4 | 2 |
| 1.6.3 | Aminophosphonate metabolism | 2 | 1 |
| 1.6.4 | Selenoamino acid metabolism | 8 | 5 |
| 1.6.5 | Cyanoamino acid metabolism | 3 | 1 |
| 1.6.7 | D-Arginine and D-ornithine metabolism | 3 | 1 |
| 1.6.9 | Glutathione metabolism | 11 | 5 |
| 1.7 Glycan Biosynthesis and metab |  |  |  |
| 1.7.2 | N-Glycans biosynthesis | 3 | 2 |
| 1.7.7 | Chondroitin / Heparan sulfate biosynthesis | 1 | 1 |
| 1.7.8 | Keratan sulfate biosynthesis | 0 | 0 |
| 1.7.9 | Lipopolysaccharide biosynthesis | 4 | 3 |
| 1.7.10 | Peptidoglycan biosynthesis | 1 | 1 |
| 1.8 Metab of Cofactors and vitamins |  |  |  |
| 1.9.1 | Ubiquinone biosynthesis | 5 | 2 |
| 1.9.2 | One carbon pool by folate | 6 | 4 |
| 1.9.4 | Riboflavin metabolism | 1 | 1 |
| 1.9.5 | Vitamin B6 metabolism | 3 | 3 |
| 1.9.6 | Nicotinate and nicotinamide metabolism | 1 | 1 |
| 1.9.7 | Pantothenate and CoA biosynthesis | 4 | 3 |
| 1.9.9 | Folate biosynthesis | 1 | 1 |
| 1.9.11 | Porphyrin and chlorophyll metabolism | 25 | 9 |
| 1.9 Biosyn of Secondary metabolites |  |  |  |
| 1.10.5 | Streptomycin biosynthesis | 2 | 2 |
| 1.10.6 | Erythromycin biosynthesis | 4 | 2 |
| 1.10.7 | Terpenoid biosynthesis | 3 | 3 |
| 1.10.8 | Flavonoids, stilbene and lignin biosynthesis | 13 | 5 |
| 1.10.9 | Alkaloid biosynthesis I | 2 | 1 |
| 1.10.10 | Alkaloid biosynthesis II | 6 | 1 |
| 1.10 Biodegredation of Xenobiotics |  |  |  |
| 1.11.2 | gamma-Hexachlorocyclohexane degradation | 1 | 1 |
| 1.11.3 | Benzoate degradation via hydroxylation | 1 | 1 |
| 1.11.7 | Tetrachloroethene degradation | 4 | 1 |
| 1.11.13 | Benzoate degradation via CoA ligation | 1 | 1 |
| 1.11.16 | Styrene degradation | 2 | 2 |
| 1.11.18 | Caprolactam degradation | 2 | 1 |
|  | **Genetic Information Processing** |  |  |
| 2.1 Transcription |  |  |  |
| 2.1.1 | RNA polymerase | 3 | 1 |
| 2.2 Translation |  |  |  |
| 2.2.3 | Aminoacyl-tRNA biosynthesis | 6 | 5 |
| 2.3 Sorting and Degredation |  |  |  |
| 2.3.2 | Type II secretion system | 2 | 2 |
| 2.3.3 | Type III secretion system | 18 | 1 |
| 2.3.6 | Ubiquitin mediated proteolysis | 9 | 1 |
| 2.3.7 | Proteasome | 5 | 1 |
| 2.4 Replication and Repair |  |  |  |
| 2.4.2 | Replication complex | 2 | 1 |
| 2.4.3 | Other factors | 2 | 1 |
|  | **Environmental Information Processing** |  |  |
| 3.2 Signal Transduction |  |  |  |
| 3.2.1 | Two-component system | 1 | 1 |
| 3.2.4 | Phosphatidylinositol signaling system | 2 | 2 |
| 3.6.1 | ATPases | 3 | 2 |
|  | **Cellular Processes** |  |  |
| 4.1 Cell motility |  |  |  |
| 4.1.2 | Flagellar assembly | 18 | 1 |
| 4.2 Cell Growth and Death |  |  |  |
| 4.2.1 | Cell cycle | 19 | 3 |
| 4.5 Behavior |  |  |  |
| 4.5.1 | Circadian rhythm | 9 | 1 |
|  | **KEGG categories not represented** |  |  |
|  | **Metabolism** |  |  |
| 1.1 | Ascorbate and aldarate metabolism | 0 | 0 |
| 1.3 | C21-Steroid hormone metabolism | 0 | 0 |
|  | Androgen and estrogen metabolism | 0 | 0 |
| 1.5 | Urea cycle and metabolism of amino groups | 0 | 0 |
| 1.6 | D-Glutamine and D-glutamate metabolism | 0 | 0 |
|  | D-Alanine metabolism | 0 | 0 |
| 1.7 | O-Glycans biosynthesis | 0 | 0 |
|  | N-Glycan degradation | 0 | 0 |
|  | Glycosaminoglycan degradation | 0 | 0 |
|  | Keratan sulfate biosynthesis | 0 | 0 |
|  | GPI-anchor biosynthesis | 0 | 0 |
|  | Sphingophospholipid biosynthesis | 0 | 0 |
|  | Sphingoglycolipid metabolism | 0 | 0 |
|  | Blood group glycolipid biosynthesis | 0 | 0 |
|  | Globoside metabolism | 0 | 0 |
|  | Ganglioside biosynthesis | 0 | 0 |
| 1.8 | Thiamine metabolism | 0 | 0 |
|  | Biotin metabolism | 0 | 0 |
|  | Retinol metabolism | 0 | 0 |
| 1.9 | Puromycin biosynthesis | 0 | 0 |
|  | Tetracycline biosynthesis | 0 | 0 |
|  | Penicillins and cephalosporins biosynthesis | 0 | 0 |
|  | Clavulanic acid biosynthesis | 0 | 0 |
|  | Indole and ipecac alkaloid biosynthesis | 0 | 0 |
|  | 1,1,1-Trichloro-2,2-bis(4-chlorophenyl)ethane (DDT) degradation | 0 | 0 |
| 1.1 | Biphenyl degradation | 0 | 0 |
|  | Toluene and xylene degradation | 0 | 0 |
|  | 2,4-Dichlorobenzoate degradation | 0 | 0 |
|  | Nitrobenzene degradation | 0 | 0 |
|  | 1,4-Dichlorobenzene degradation | 0 | 0 |
|  | Fluorene degradation | 0 | 0 |
|  | Carbazole degradation | 0 | 0 |
|  | 1,2-Dichloroethane degradation | 0 | 0 |
|  | 3-Chloroacrylic acid degradation | 0 | 0 |
|  | Ethylbenzene degradation | 0 | 0 |
|  | Atrazine degradation | 0 | 0 |
|  | **Genetic Information Processing** |  |  |
| 2.1 | Basal transcription factors | 0 | 0 |
|  | HTH family transcriptional regulators | 0 | 0 |
|  | Other and unclassified family transcriptional regulators | 0 | 0 |
|  | Spliceosome | 0 | 0 |
| 2.2 | Ribosome | 0 | 0 |
|  | Translation factors | 0 | 0 |
|  | Other translation factors | 0 | 0 |
| 2.3 | Protein export | 0 | 0 |
|  | Type IV secretion system | 0 | 0 |
| 2.4 | RNA degradosome | 0 | 0 |
|  | DNA polymerase | 0 | 0 |
|  | **Environmental Information Processing** |  |  |
| 3.1 | ABC transporters, prokaryotic | 0 | 0 |
|  | ABC transporters, ABC-2 and other types | 0 | 0 |
|  | ABC transporters, eukaryotic | 0 | 0 |
|  | Major facilitator superfamily (MFS) | 0 | 0 |
|  | Other ion-coupled transporters | 0 | 0 |
|  | Pores ion channels | 0 | 0 |
|  | Other transporters | 0 | 0 |
| 3.2 | Phosphotransferase system (PTS) | 0 | 0 |
|  | MAPK signaling pathway | 0 | 0 |
|  | Second messenger signaling pathway | 0 | 0 |
| 3.3 | G-protein coupled receptors | 0 | 0 |
|  | Ion channel receptors | 0 | 0 |
|  | **Cellular Processes** |  |  |
| 4.2 | Apoptosis | 0 | 0 |
| 4.3 | Integrin-mediated cell adhesion | 0 | 0 |
|  | Cadherin-mediated cell adhesion | 0 | 0 |
| 4.4 | Wnt signaling pathway | 0 | 0 |
|  | Notchsignaling pathway | 0 | 0 |
|  | Dorso-ventral axis formation | 0 | 0 |
|  | **KEGG Categories Eliminated** |  |  |
| 5.1 | **Human Diseases** |  |  |
